# Bibliometric analysis of global research on PD-L1/PD-1 pathway and neurodegenerative diseases over the last two decades (2004–2023)

**DOI:** 10.3389/fnagi.2025.1570428

**Published:** 2025-05-30

**Authors:** Jialin Wu, Chaojin Zhang, Qiang Cao, Wei Xuan, Xiaorong Huai, Jie Zhou, Jie Tian

**Affiliations:** ^1^Department of Anesthesiology, Renji Hospital, Shanghai Jiao Tong University School of Medicine, Shanghai, China; ^2^Key Laboratory of Anesthesiology (Shanghai Jiao Tong University), Ministry of Education, Shanghai, China

**Keywords:** PD-1, PD-L1, neurodegenerative diseases, bibliometric analysis, CiteSpace, VOSviewer

## Abstract

**Background:**

Programmed death receptor 1 (PD-1), encoded by the PDCD1 gene, functions as a pivotal immunosuppressive molecule. Neurodegenerative diseases (NDDs) encompass a diverse array of neurological disorders that adversely impact the lives of millions of individuals globally. The current study discusses the impacts of PD-L1/PD-1 signaling on NDDs.

**Methods:**

A comprehensive online search was conducted using the Web of Science Core Collection database (WOSCC), with a limited time frame set from 2004 to 2023. Data were analyzed with CiteSpace, VOSviewer, and bibliometric package to explore trends in research output, key authors, institutions, journals, and thematic developments.

**Results:**

This study analyzed 366 publications within the field of PD-L1/PD-1 and NDDs. During 2004–2023, there’s an overall upward trajectory in the number of publications as the years progressed. The United States has a significant influence in this field, accounting for the highest number of publications. It also boasts the top two authors, six of the top 10 journals, and four of the top five institutions in terms of article count. Keyword burst analysis identified EAE, Parkinson’s disease, adaptive immunity, immune checkpoint blockade, and cerebrospinal fluid are research hotspots in recent years.

**Conclusion:**

This field has garnered increasing research attention, with the United States being the primary contributor. Recent studies have concentrated on the mechanisms through which PD-L1/PD-1 influences NDDs, and research into cerebrospinal fluid may persist as a focal point in the years to come. While the neuroprotective vs. neurodegenerative effects of PD-L1/PD-1 signaling remain controversial, this pathway represents a promising diagnostic and therapeutic target for NDDs.

## 1 Introduction

Programmed death receptor 1 (PD-1), encoded by the PDCD1 gene, serves as a key immunosuppressive molecule. Its expression extends beyond conventional T cells to regulatory T cells, B cells, natural killer (NK) cells, activated monocytes, and dendritic cells (DCs) (Sharpe and Pauken, 2018). When bound to its ligands, PD-L1 or PD-L2, PD-1 inhibits T cell activation (Bour-Jordan et al., 2011; Tan et al., 2021). These immunomodulatory properties have made PD-1 a major research focus in oncology, driving the development of PD-1 pathway inhibitors for various cancer treatments (Borghaei et al., 2015; Garon et al., 2015; Motzer et al., 2015; Callahan et al., 2016; Ferris et al., 2016).

Neurodegenerative diseases (NDDs) are characterized by the progressive degeneration of neurons or myelin sheaths within the central nervous system (CNS) and the peripheral nervous system (PNS). The limited regenerative capacity of neurons results in declining memory, cognition, behavior, sensory perception, and/or motor function (Dugger and Dickson, 2017). Numerous studies have confirmed a correlation between the PD-L1/PD-1 pathway and NDDs (Rosenzweig et al., 2019; Tsaktanis et al., 2023; Zhao et al., 2023), though its precise roles remain unclear.

NDDs comprise Alzheimer’s disease (AD), Parkinson’s disease (PD), primary tauopathies, frontotemporal dementia, amyotrophic lateral sclerosis (ALS), synucleinopathies, Huntington’s disease, polyglutamine diseases (including spinocerebellar ataxias), prion disease, traumatic brain injury, chronic traumatic encephalopathy, stroke, spinal cord injury, and multiple sclerosis (MS). The most recent research has established a framework for unifying and categorizing NDDs, encompassing pathological protein aggregation, synaptic and neuronal network dysfunction, aberrant proteostasis, cytoskeletal abnormalities, altered energy metabolism, DNA and RNA defects, inflammation, and neuronal cell death (Wilson et al., 2023). Based on this framework, the present study utilizes bibliometric methods to classify, summarize, and visualize the literature pertaining to PD-L1/PD-1 and NDDs published between 2004 and 2023. Furthermore, it discusses the impacts of PD-L1/PD-1 on the NDDs, serving as a valuable reference for future researchers.

## 2 Methods

### 2.1 Data source and search strategy

A comprehensive online search was conducted using the Web of Science Core Collection database (WOSCC), with a limited time frame set from 2004 to 2023. The employed search strategy is outlined below: TS = ((“PD-1” OR “PD-L1” OR “PD 1” OR “PD L1” OR “PD1” OR “PDL1” OR “programmed cell death 1” OR “programmed cell death 1 ligand 1” OR (PDCD1) OR (PDCD1 ligand 1)) AND ((Central degenerative lesions) OR (neurodegenerative diseases) OR (Alzheimer’s disease) OR (Parkinson’s disease) OR (Huntington’s disease) OR (Amyotrophic lateral sclerosis) OR (Spinocerebellar ataxia) OR (Pick’s disease) OR (epilepsy) OR (tauopathy) OR (Alzheimer disease) OR (Parkinson disease) OR (primary tauopathies) OR (frontotemporal dementia) OR (amyotrophic lateral sclerosis) OR (synucleinopathies) OR (Lewy body dementia) OR (multisystem atrophy) OR (Huntington disease) OR (related polyglutamine (polyQ) diseases) OR (including spinocerebellar ataxias) OR (prion disease) OR (traumatic brain injury) OR (chronic traumatic encephalopathy) OR (stroke) OR (spinal cord injury) OR (multiple sclerosis) OR (amyloid precursor protein))) NOT TI = ((guideline) OR (recommendation) OR (consensus) OR (case report) OR (meta) OR (review)).

**FIGURE 1:**
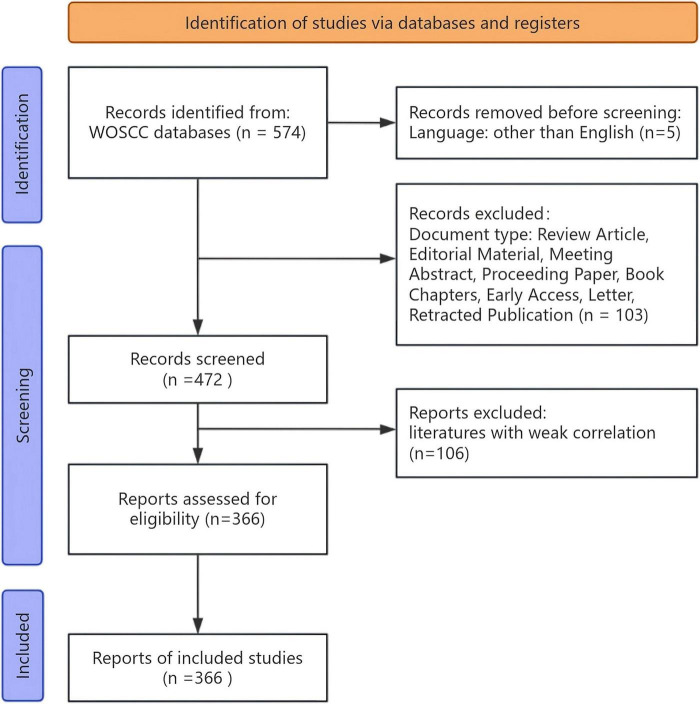
Workflow diagram of documents searching phase and the result of each stage in this study.

A total of 574 articles were retrieved initially. After excluding review articles and publications with low relevance to ensure study quality, we identified 366 eligible articles (Figure 1). All literature searches and data from these 366 studies were downloaded on June 10, 2024, ensuring a consistent and up-to-date dataset for further analysis.

### 2.2 Data analysis

The full records and cited references of the publications were downloaded from the WOSCC database and saved in.txt format. This study employed two software tools and a R-package, for constructing and visualizing bibliometric networks. The impact factors (IFs) of the journals were retrieved from the 2023 Journal Citation Reports (JCR), published by Clarivate Analytics, Philadelphia, United States.

The data from the WOSCC database were imported into CiteSpace software v6.3R1, 64-bit (Drexel University, Philadelphia, PA, United States), utilizing the following settings: a time slicing range from January 2004 to December 2023, with 1 year per slice. A modified g-index was applied within each slice, defined as g^2^ ≤ kΣ_*i* = *g*_ c_*i*_, k ∈ Z^+^, k = 25. Network maps were generated using CiteSpace, where nodes represented various elements such as country, institution, and keyword, with node size indicating the number of publications or frequency. The connections between nodes represented relationships such as collaboration, co-occurrence, or co-citation. Node and line colors denoted different clusters or time periods. For the analysis of national, institutional, or co-authorship networks, “Country,” “Institution,” or “Author” was selected in the Node Types parameter section, with the remaining settings set to their default values. For keyword burst detection, “Keywords” was chosen as the Node Type, and “Cosine” was employed to calculate burst strength. Following the removal of keywords with minimal practical significance (e.g., cells, mice), the top 14 keywords with the highest burst strength were identified and displayed in WPS Office.

VOSviewer version 1.6.20 (Leiden University, Leiden, Netherlands) was utilized to analyze the publication years and to design a network map for the purpose of keyword cluster analysis. Upon importing the data into VOSviewer, “Co-occurrence” and “All keywords” options were selected, subsequently facilitating the classification of keywords into distinct clusters.

Bibliometric package (Aria and Cuccurullo, 2017) is a powerful and versatile tool specifically designed for bibliometric analysis. It is used to create national cooperation maps and analyze the evolution of keywords.

## 3 Results

### 3.1 Publication outputs

**FIGURE 2:**
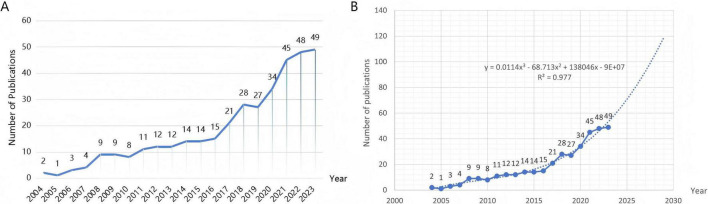
**(A)** The annual publication counts and growth trends in the field of PD-L1/PD-1 and NDDs from 2004 to 2023. The data were exported from VOSviewer and plotted using WPS software. The numerical values located above the line represent the annual number of publications. **(B)** The growth trend model built by Microsoft Excel 2021.

This study analyzed 366 publications within the field of PD-L1/PD-1 and NDDs, spanning the period from 2004 to 2023. A line chart was employed to illustrate the trend in the number of publications over the years. The results demonstrated an overall upward trajectory in the number of publications as the years progressed (Figure 2A). Notably, the substantial increase observed between 2016 and 2023 suggests a growing interest in this research area. Furthermore, a growth trend model was built using Microsoft Excel 2021 as follows: f(x) = ax^3^ + bx^2^ + cx + d, which indicated that the number of articles in 2026 will exceed 80 (Figure 2B). This may be related to the impetus provided by immunotherapeutic approaches. Researchers have begun to focus on the application of immunotherapies in fields other than cancer (Qin et al., 2019).

### 3.2 Countries/regions and institutions

**FIGURE 3:**
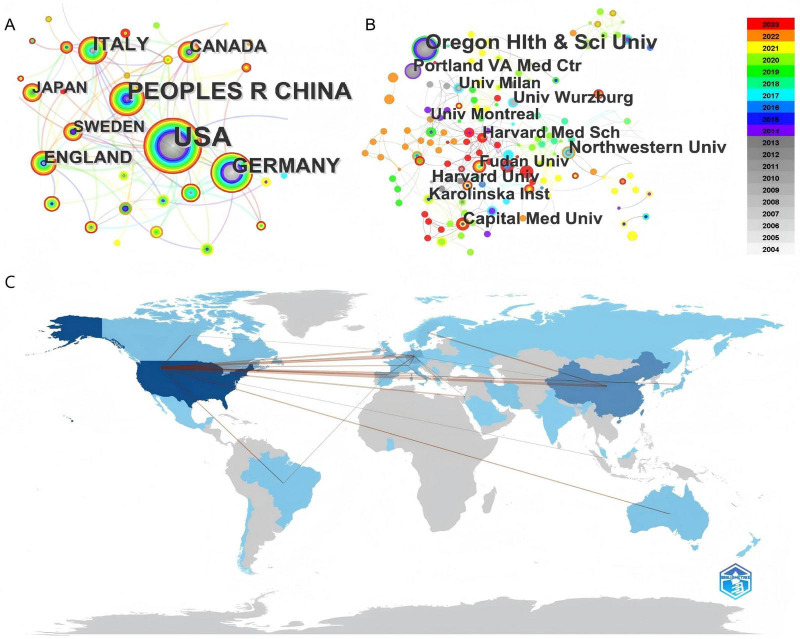
**(A,B)** The network map of countries Country/regional **(A)** and institutions **(B)** in the field of NDDs related to PD-L1/PD-1 research. The results are exported from CiteSpace. The size of each circle corresponds to the number of publications, the connecting lines represent cooperative efforts between them, and various colors denote different years. **(C)** The map of international cooperation, made by bibliometric R-package. The line represents the cooperation between the two countries, and the thickness represents the frequency of cooperation.

To investigate the number of publications in the field of PD-L1/PD-1 and NDDs across various countries/regions or institutions, further analysis of publications in different countries/regions or institutions was conducted using CiteSpace (Figures 3a,b). The size of each circle indicates the number of publications, the links represent collaborations between them, and different colors denote different years.

The publications originated from 40 countries, with the United States contributing the largest number, totaling 148 articles over the past 20 years. China ranked second with 74 articles, followed by Germany (40 articles), Italy (23 articles), and the United Kingdom (18 articles). Temporal analysis revealed sustained leadership by the US and Germany throughout the study period, while China, Italy and the UK showed accelerated productivity post-2017 (Figure 3A). The map of international cooperation more vividly illustrates the cooperative relationships among countries worldwide. It is evident that there is extensive collaboration between countries globally, with the United States engaging in the most frequent partnerships with other countries (Figure 3C).

A total of 663 institutions participated in this study. The top five institutions with the highest publication counts are: Oregon Health and Science University (22 articles), Northwestern University (17 articles), Capital Medical University (12 articles), Harvard University (12 articles), and Portland VA Medical Center (12 articles). However, despite leading in total publications, the majority of research at these institutions was conducted in earlier years (Figure 3B). Conversely, although Fudan University and Capital Medical University have a relatively smaller total number of publications, their research has primarily been conducted in recent years.

### 3.3 Journals and high-cited articles

A total of 366 papers were published across 200 journals. Table 1 presents the top 10 most cited journals, ranked by total citations. The *Journal of Immunology* stands at the top with a total of 953 citations, averaging 56.06 citations per paper. Among these, *Nature Medicine* boasts the highest impact factor, according to the 2023 JCR. Notably, eight out of the top 10 journals are categorized in Q1, with the exceptions of *Journal of Immunology* and *Journal of Neuroimmunology*, which are ranked in Q2 (Table 1).

**TABLE 1:** Top 10 active journals with the highest number of total citations that published articles in PD-L1/PD-1 and NDDs research (sorted by count).

Rank	Journal title	Number of publications	Citations	Average citation per paper	Impact factor (2023)	Country	JCR
1	Journal of Immunology	17	953	56.06	3.6	United States	Q2
2	Journal of Neuroimmunology	17	752	44.24	2.9	Netherlands	Q2
3	Frontiers in Immunology	26	539	20.73	5.7	Switzerland	Q1
4	Nature Communications	4	423	105.75	14.7	England	Q1
5	Nature Medicine	3	377	125.67	58.7	United States	Q1
6	Journal of Neuroinflammation	24	348	14.50	9.3	England	Q1
7	Blood	2	298	149.00	21.0	United States	Q1
8	Annals of Neurology	2	286	143.00	8.1	United States	Q1
9	Stroke	6	278	46.33	7.8	United States	Q1
10	Cell	1	277	277	45.5	United States	Q1

Table 2 outlines the top 10 highly cited articles, evaluated based on the total number of citations across all fields. The research article titled “Early detrimental T-cell effects in experimental cerebral ischemia are neither related to adaptive immunity nor thrombus formation,” published in Blood by Kleinschnitz et al. (2010), received the highest number of citations. Its main research findings indicate that T cells critically contribute to cerebral ischemia, but their detrimental effect neither depends on antigen recognition nor T-cell receptor (TCR) costimulation or thrombus formation. This is followed by “PD-1 immune checkpoint blockade reduces pathology and improves memory in mouse models of Alzheimer’s disease,” which indicates that immune checkpoint blockade is a novel therapeutic strategy for AD and, potentially, for other neurodegenerative diseases. It was published in Nature Medicine by Baruch et al. (2016), with 265 citations.

**TABLE 2:** Top 10 high-cited articles in PD-L1/PD-1 and NDDs research during the years 2004–2023.

Number	Authors	Years	Journal	Total citations	Title	Core Contribution
1	Kleinschnitz, C	2010	Blood	280	Early detrimental T-cell effects in experimental cerebral ischemia are neither related to adaptive immunity nor thrombus formation	T cells critically contribute to cerebral ischemia, but their detrimental effect neither depends on antigen recognition nor TCR costimulation or thrombus formation.
2	Baruch, K	2016	Nature Medicine	265	PD-1 immune checkpoint blockade reduces pathology and improves memory in mouse models of Alzheimer’s disease	Immune checkpoint blockade s a novel therapeutic strategy for AD and, potentially, for other neurodegenerative diseases.
3	Mokarizadeh, A	2012	Immunology Letters	249	Microvesicles derived from mesenchymal stem cells: potent organelles for induction of tolerogenic signaling	The results described in this study introduce, for the first time, a new approach to peripheral tolerance induction via biological intervention in immune system using mesenchymal stem cells-derived microvesicles as novel attractive tolerogenic organelles for treatment and control of autoimmune diseases.
4	Schreiner, B	2004	Journal of Neuroimmunology	232	Interferon-β enhances monocyte and dendritic cell expression of B7-H1 (PD-L1), a strong inhibitor of autologous T-cell activation: relevance for the immune modulatory effect in multiple sclerosis	B7-H1 expressed on APC acts as a strong inhibitor of autologous CD4 T-cell activation and may thus contribute to the maintenance of peripheral immune tolerance. IFN-β up-regulates B7-H1 *in vitro* and in MS patients *in vivo* and might represent a novel mechanism how IFN-β acts as a negative modulator on APC T-cell interactions in the periphery.
5	Smolders, J	2018	Nature Communications	214	Tissue-resident memory T cells populate the human brain	The human brain is surveilled by TRM cells, providing protection against neurotropic virus reactivation, whilst being under tight control of key immune checkpoint molecules.
6	Kroner, A	2005	Annals of Neurology	193	A PD-1 polymorphism is associated with disease progression in multiple sclerosis	PD-1 polymorphism is a genetic modifier of the progression of MS, possibly through inducing a partial defect in PD-1–mediated inhibition of T-cell activation.
7	Getts, DR	2011	Journal of Immunology	158	Tolerance induced by apoptotic antigen-coupled leukocytes is induced by PD-L1 + and IL-10-producing splenic macrophages and maintained by T regulatory cells	Experimental autoimmune encephalomyelitis, High-density-lipoprotein, dendritic cells, self-tolerance, peripheral tolerance, marginal zone, B-cells, SR-BII, induction, mice
8	Carter, LL	2007	Journal of Neuroimmunology	149	PD-L1/PD-1, but not PD-1/PD-L2, interactions regulate the severity of experimental autoimmune encephalomyelitis	Interactions between PD-1/PD-L1, but not PD-1/PDL-2, are crucial in attenuating T cell responses in EAE.
9	Karni, A	2006	Journal of Immunology	135	Innate immunity in multiple sclerosis: myeloid dendritic cells in secondary progressive multiple sclerosis are activated and drive a proinflammatory immune response	The secondary progressive form of MS may have a T cell-independent component and that therapies that target myeloid dendritic cells and the innate immune system may be effective in this stage of the disease.
10	Wang, C	2009	Immunology	120	Estrogen modulates experimental autoimmune encephalomyelitis and interleukin-17 production via programmed death 1	17 β-estradiol-induced protection against EAE is mediated by upregulation of PD-1 expression within the Treg-cell compartment.

It is noteworthy that all the most influential articles were published in earlier years, and none of the top 10 highly cited articles appear within the last 5 years. This suggests that the field has not witnessed significant advancements in recent times, indicating ample opportunities for further development.

### 3.4 Authors and co-cited authors

**FIGURE 4:**
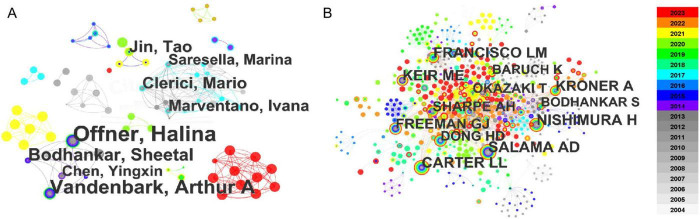
The network map of authors **(A)** and co-cited authors **(B)** for PD-L1/PD-1 research in the NDDs field. The results are exported from CiteSpace. The diameter of the circle signifies the volume of publications or the citation count, while the links depict the cooperative or co-cited relationships among them. Various colors denote different years.

Figure 4A illustrates the number of publications by authors in the fields of PD-1 signaling and NDDs, as well as the collaborations among these authors. Each circle represents an individual author, with the circle’s size directly proportional to the number of publications. The connections between circles signify author collaborations, therefore clusters of interconnected circles represent cooperative groups. Between 2004 and 2023, a total of 2,680 authors contributed to the field of PD-L1/PD-1 and NDDs. Halina Offner led with 21 publications, followed closely by Arthur A. Vandenbark with 15 articles (Figure 4A). Notably, the majority of their publications preceded 2018, and a collaborative relationship existed between the two authors. Author groups that collaborate at different times are differentiated by color. A dense cluster of red circles in the lower right corner, with numerous interconnections, signifies a collaboration hub in 2023, indicating frequent collaboration among these authors and their prominent role in recent research endeavors (Figure 4A).

Core authors are a select few within a discipline or professional field who exhibit a high citation rate. To identify core authors, a co-cited authorship analysis was conducted using CiteSpace, yielding 908 nodes and 3,476 links (Figure 4B). The size of each circle represents the citation count, while the links depict co-citation relationships. Different colors represent distinct years. Alan D. Salama of University College London Medical School emerged as the top-cited author. The remaining prominent authors, whose articles have been widely cited between 2004 and 2023, are presented in Figure 3B.

### 3.5 Cluster analysis and burst detection with keywords

**FIGURE 5:**
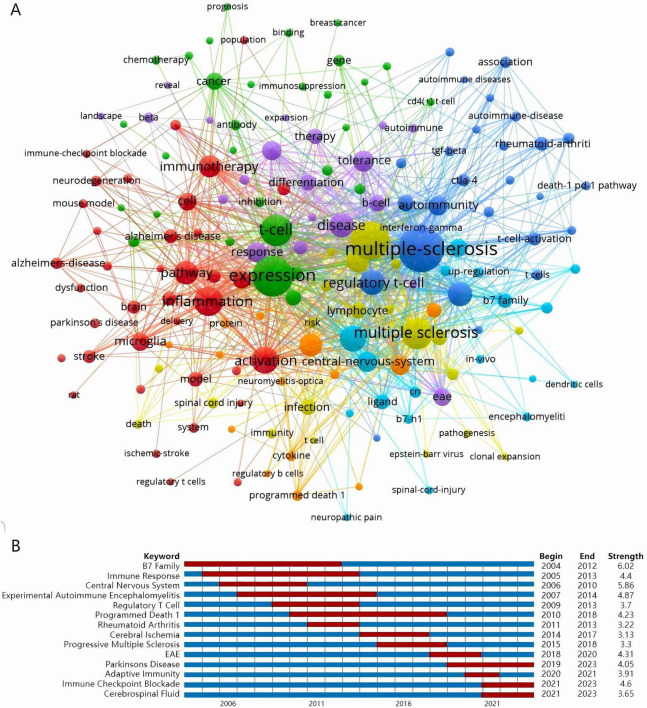
**(A)** Network map of keyword clustering showing keywords with a minimum occurrence of five times, classified into seven clusters: inflammation, expression, multiple-sclerosis, PD-1, experimental autoimmune encephalomyelitis (EAE), PD-L1, central-nervous-system. The results are exported from VOSviewer. The diameter of the circles signifies the frequency of occurrence of the keywords, while the lines indicate co-occurrence within the same article. Different colors represent distinct categories. **(B)** Keywords with the strongest citation bursts in original articles on PD-L1/PD-1 and NDDs research between 2004 and 2023. Keywords marked in red indicates a sudden increase in the usage frequency of this keyword during that period. Blue represents a relatively unpopular time period. The data was exported by CiteSpace and the graph was made by WPS software.

Keyword clustering and burst detection techniques provide insights into the evolutionary trends of research within a specific field, facilitating a better understanding of the prevalent research hotspots. By extracting keywords from 366 articles, 167 keywords appeared at least five times. VOSviewer was utilized to cluster and visualize these keywords, resulting in seven categories: inflammation, expression, multiple-sclerosis, PD-1, experimental autoimmune encephalomyelitis (EAE), PD-L1, central-nervous-system (Figure 5A). The diameter of the circles signifies the frequency of occurrence of the keywords, while the lines indicate co-occurrence within the same article. Different colors represent distinct categories. Table 3 displays the quantity of keywords in each cluster, as well as the top three keywords with the highest frequency of occurrence.

**TABLE 3:** Keywords in clusters: quantity and top—three high-frequency list.

Clusters	Quantity	Keywords
1	37	Inflammation, activation, pathway
2	30	Expression, T-cell, cancer
3	27	Regulatory T-cell, experimental autoimmune encephalomyelitis, autoimmunity
4	21	Multiple-sclerosis, PD-1, multiple sclerosis
5	20	Disease, tolerance, response
6	18	PD-L1, central-nervous-system, induction
7	14	Mice, experimental autoimmune encephalomyelitis, programmed death-1

Furthermore, a burst word analysis of the 366 articles over the past 20 years was conducted using CiteSpace. Figure 5B illustrates the range of years examined for citation burst durations represented by red areas. After excluding keywords with minimal or no research significance, we were left with 14 keywords. The burst detection analysis revealed that topics such as B7 family, immune response, central nervous system, regulatory T cells, and rheumatoid arthritis were prevalent before 2014. In contrast, recent hot topics include EAE, Parkinson’s disease, adaptive immunity, immune checkpoint blockade, and cerebrospinal fluid. The emergence of these topics as research hotspots likely stems from the progressive elucidation of immunoregulatory mechanisms underlying NDDs in recent years. This temporal progression has witnessed a paradigm shift in scientific inquiry, particularly toward deciphering the neuroimmunological axis mediated by PD-L1/PD-1 signaling pathways across heterogeneous pathological contexts (Duffy et al., 2014; Zhuo et al., 2023). These thematic elements are projected to maintain their centrality in forthcoming investigative endeavors.

### 3.6 Evolution of key words

**FIGURE 6:**
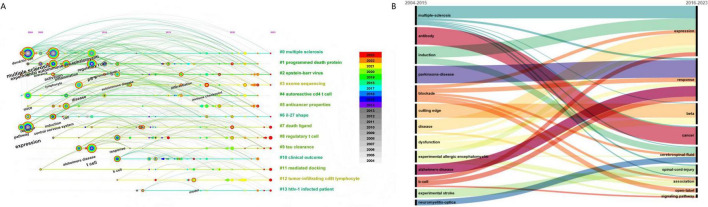
**(A)** Time Zone View of keywords in the field of PD-L1/PD-1 and NDDs from 2004 to 2023. The results are exported from CiteSpace. The horizontal axis signifies various clusters, with the position of the circle indicating the time of the keyword’s initial appearance, the color of the circle denoting different years, and the lines emanating from them showing that these keywords were revisited for further study. **(B)** Sankey diagram with 2016 as the central node. The data were obtained by bibliometric R-package and made by RAWGraphs 2.0.

The Time Zone View facilitates the visualization of research hotspot evolution within the PD-L1/PD-1 and NDDs domain, while also enabling the prediction of future research trends. Over the past two decades, MS, EAE, along with the CNS, have been extensively researched. In contrast, investigations into differentiation have only emerged more recently (Figure 6A).

Given the inflection point marked by the 2016 surge in publication output, a Sankey diagram was constructed to visualize keyword evolution dynamics (Figure 6B). Initial research endeavors (pre-2016 phase) were principally devoted to nosological characterization of MS, PD, and EAE. Subsequent investigations (post-2016) have systematically transitioned toward elucidating transcriptional regulatory networks and downstream molecular responses of these disorders.

## 4 Discussion

The present study visualized research articles in the field of PD-L1/PD-1 and NDDs spanning from 2004 to 2023. The research output in this field has demonstrated a sustained growth trajectory, with notably increased scholarly attention in recent years. Our research employs a diverse array of methodologies, including the analysis of authors, countries, journals, keywords, and other facets, to illustrate the current research landscape of this field and provide guidance for future endeavors.

Among the countries participating in research on PD-L1/PD-1 and NDDs, the United States stands out prominently. The country not only maintains the highest publication volume but also dominates key academic metrics: it is home to the two most prolific authors, hosts six of the top 10 publishing journals, and contains three of the top five research institutions by output. Other countries have also been thriving and making substantial contributions. However, the analysis of highly cited articles indicates that despite the rapid expansion of research output in the field of PD-L1/PD-1 and NDDs, only a limited number of influential articles have been published in recent years, suggesting vast areas remain ripe for exploration and development.

The detection of keyword bursts signifies emerging trends in the research domain of PD-L1/PD-1 and NDDs. In recent years, EAE, Parkinson’s disease, adaptive immunity, immune checkpoint blockade, and cerebrospinal fluid have garnered significant research attention. Notably, cerebrospinal fluid studies may represent a promising direction for future research. Currently, preliminary insights into the mechanisms by which PD-L1/PD-1 contribute to NDDs have been obtained. While substantial data supports hypotheses regarding their potential impact on the progression of NDDs, the precise role of PD-L1/PD-1 signaling remains contentious. The fundamental question of whether PD-L1/PD-1 exerts neuroprotective or neurotoxic effects in the context of NDDs continues to be debated within scientific community.

Alterations in the expression of PD-L1/PD-1 have been observed in NDDs, both centrally and peripherally (Braun et al., 2023). Studies have demonstrated that PD-1 and PD-L1 are upregulated in the CNS of mice with demyelinating disease induced by Theiler’s murine encephalomyelitis virus (Jin et al., 2013; Takizawa et al., 2014). Similarly, elevated levels of secreted PD-1 (sPD-1) and secreted PD-L1 (sPD-L1) were also observed in the serum of patients with ALS and relapsing-remitting multiple sclerosis (RRMS) (Beers et al., 2021; Tsaktanis et al., 2023). These alterations frequently coincide with an exacerbation of neuroinflammation or a deterioration in disease condition.

PD-1 and PD-L1 are expressed in multiple immune cell populations, including T cells, B cells, NK cells, activated monocytes, DCs, as well as in CNS-resident microglia and astrocytes, where they play pivotal roles in modulating both innate and adaptive immunity (Sharpe and Pauken, 2018; Tan et al., 2021; Linnerbauer et al., 2023). In addition to its expression in immune cells, PD-1 is also present in CNS neurons, exerting an influence on their function. The gene encoding PD-1, PDCD1, is expressed specifically in cortical and thalamic neurons. When the PDCD1 gene is knocked down in mice, they exhibit enhanced learning and memory capabilities, as well as increased neuronal excitability and dendritic spine density (Zhao et al., 2023). Following the loss of PD-1, excessive neuronal activity ensued due to ERK-mediated suppression of A-type potassium channels, and the ERK-mediated enhancement of NMDAR and AMPAR activity in neurons may be responsible for synaptic plasticity. However, during the early stages of neural development, the absence of PD-1 exacerbates neuronal damage. PD-1 is abundantly expressed on the cell membrane of neural progenitors and plays a crucial role in neuronal migration and differentiation. This effect is mediated by Pax3 through AKT/GSK-3β/β-catenin signaling pathway. Ablation of PD-1 leads to impaired embryonic neurogenesis, characterized by accelerated proliferation of neural progenitors, reduced neural differentiation, and delayed neuronal migration (Ji et al., 2023). These findings indicate that the PD-L1/PD-1 pathway exhibits differential and even opposing effects on neurons at various developmental stages.

This dual regulatory effect is similarly manifested in neuroinflammatory processes. Javan et al. (2016) demonstrated reduced PD-L1/PD-1 expression in peripheral blood mononuclear cells from RRMS patients, contrasting with the elevated levels reported by Tsaktanis et al. (2023). Although the etiological factors vary considerably among different NDDs, progressive neuronal degeneration and myelin sheath loss are consistently associated with chronic innate immune activation (Stephenson et al., 2018). Within the CNS, microglia and astrocytes serve as the principal innate immune effectors, and their sustained activation along with consequent neuroinflammation have been established as pathological hallmarks of NDDs (Heneka et al., 2014). During chronic neuroinflammation, interferon-gamma (IFN-γ) stimulates PD-L1 upregulation in astrocytes, which subsequently induces microglial M2 polarization and suppresses proinflammatory activation through STAT3 signaling pathway activation, thereby attenuating CNS autoimmunity (Linnerbauer et al., 2023; Wang et al., 2024). These findings collectively suggest a neuroprotective role of PD-L1/PD-1 signaling. Inhibiting this pathway in astrocytes and microglia can lead to microglia polarization, exacerbating neuroinflammation and ultimately disease progression, which further supports this point (Takizawa et al., 2014; Yao et al., 2014; Wu et al., 2017; Cheng et al., 2022). Nevertheless, the precise mechanisms underlying PD-L1/PD-1-mediated neuroinflammatory suppression remain elusive, particularly regarding the clinical significance of pathway upregulation in RRMS and its potential disease-stage dependency. In this context, future studies will be required to elucidate the disease-stage-specific role of PD-L1/PD-1 signaling.

From a therapeutic perspective, emerging evidence suggests that modulation of the PD-L1/PD-1 axis may hold promise for treating NDDs. In spinal cord injury (Yao et al., 2014), MS (Takizawa et al., 2014; Linnerbauer et al., 2023), and traumatic encephalopathy (Wu et al., 2017), therapeutic activation of this pathway represents a potential intervention strategy, although clinical validation remains lacking. Given the stage-dependent duality of PD-L1/PD-1 signaling effects, particular consideration must be given to both the intensity of pathway modulation and disease progression phases, especially in MS. In AD and tauopathy models, PD-1 blockade elicits a systemic IFN-γ-mediated immune response that recruits monocyte-derived macrophages to the brain. This leads to amyloid-β (Aβ) plaque reduction, attenuated neuropathological changes, and improved cognitive function (Baruch et al., 2016). These macrophages secrete IL-10, an anti-inflammatory agent, and directly influence astrocytes, or indirectly affect microglia, to reduce the secretion of IL-1β (Rosenzweig et al., 2019). However, since PD-1 maintains CD36 expression and promotes the uptake of aggregated Aβ by microglia, complete loss of PD-1 signaling using the PD-1 knockout model worsened the progression of plaque pathology and cognition (Kummer et al., 2021). These findings suggest a potential therapeutic strategy combining PD-L1/PD-1 pathway enhancement with concurrent suppression of other immune-related exhaustion factors.

In the last 2 years, there have been several new research developments. Notably, Mi et al. (2024) pioneered the demonstration of therapeutic efficacy of exogenous sPD-L1 supplementation on DCs in MS. Treatment with sPD-L1 downregulates CD86 expression, inhibits CCR7-mediated DCs migration, and enhances DCs phagocytic capacity. Another study attempted to block the IL-6 signal in EAE mice; however, this intervention failed to demonstrate significant clinical improvement in disease progression (Dema et al., 2024). In addition, an article have reviewed the medicinal value of astragalus polysaccharide (APS) in NDDs, including AD, PD, and MS (Shi and Ma, 2024). In MS, APS alleviates symptoms by inhibiting T cell proliferation and reducing the expression of pro-inflammatory cytokines via the PD-1/PD-Ls pathway.

## 5 Conclusion

In conclusion, over the past 2 decades, research on the PD-L1/PD-1 pathway and NDDs has made significant strides and is increasingly gaining attention. However, there remains ample room for further exploration. Ongoing research continues to generate heated debates and conflicting interpretations, and it is unclear how PD-L1/PD-1 influences NDDs at various stages. Alongside resolving these debates, exploring PD-L1/PD-1 pathway regulation offers promising therapeutic potential for neurodegenerative disorders.

## Data Availability

The original contributions presented in the study are included in the article/supplementary material, further inquiries can be directed to the corresponding authors.

## References

[B1] Aria M. Cuccurullo C. (2017). *bibliometrix*: An R-tool for comprehensive science mapping analysis. *J. Informetr.* 11 959–975. 10.1016/j.joi.2017.08.007

[B2] Baruch K. Deczkowska A. Rosenzweig N. Tsitsou-Kampeli A. Sharif A. Matcovitch-Natan O. (2016). PD-1 immune checkpoint blockade reduces pathology and improves memory in mouse models of Alzheimer’s disease. *Nat. Med*. 22 135–137. 10.1038/nm.4022 26779813

[B3] Beers D. Zhao W. Thonhoff J. Faridar A. Thome A. Wen S. (2021). Serum programmed cell death proteins in amyotrophic lateral sclerosis. *Brain Behav. Immun. Health* 12:100209. 10.1016/j.bbih.2021.100209 34589734 PMC8474632

[B4] Borghaei H. Paz-Ares L. Horn L. Spigel D. Steins M. Ready N. (2015). Nivolumab versus docetaxel in advanced nonsquamous non-small-cell lung cancer. *N. Engl. J. Med*. 373 1627–1639. 10.1056/NEJMoa1507643 26412456 PMC5705936

[B5] Bour-Jordan H. Esensten J. Martinez-Llordella M. Penaranda C. Stumpf M. Bluestone J. (2011). Intrinsic and extrinsic control of peripheral T-cell tolerance by costimulatory molecules of the CD28/ B7 family. *Immunol. Rev*. 241 180–205. 10.1111/j.1600-065X.2011.01011.x 21488898 PMC3077803

[B6] Braun M. Boström G. Ingelsson M. Kilander L. Löwenmark M. Nyholm D. (2023). Levels of inflammatory cytokines MCP-1, CCL4, and PD-L1 in CSF differentiate idiopathic normal pressure hydrocephalus from neurodegenerative diseases. *Fluids Barriers CNS* 20:72. 10.1186/s12987-023-00472-x 37833765 PMC10571396

[B7] Callahan M. Postow M. Wolchok J. (2016). Targeting T cell co-receptors for cancer therapy. *Immunity* 44 1069–1078. 10.1016/j.immuni.2016.04.023 27192570

[B8] Cheng Y. Chen B. Bian G. Ding Y. Chen L. (2022). Programmed death-1 deficiency aggravates motor dysfunction in MPTP model of Parkinson’s Disease by inducing microglial activation and neuroinflammation in mice. *Mol. Neurobiol*. 59 2642–2655. 10.1007/s12035-022-02758-x 35142987

[B9] Dema M. Eixarch H. Castillo M. Montalban X. Espejo C. (2024). IL-6 inhibition as a therapeutic target in aged experimental autoimmune encephalomyelitis. *Int. J. Mol. Sci*. 25:6732. 10.3390/ijms25126732 38928437 PMC11204061

[B10] Duffy S. Lees J. Moalem-Taylor G. (2014). The contribution of immune and glial cell types in experimental autoimmune encephalomyelitis and multiple sclerosis. *Mult. Scler. Int*. 2014:285245. 10.1155/2014/285245 25374694 PMC4211315

[B11] Dugger B. Dickson D. (2017). Pathology of neurodegenerative diseases. *Cold Spring Harb. Perspect. Biol*. 9:a028035. 10.1101/cshperspect.a028035 28062563 PMC5495060

[B12] Ferris R. Blumenschein G. Fayette J. Guigay J. Colevas A. Licitra L. (2016). Nivolumab for recurrent squamous-cell carcinoma of the head and neck. *N. Engl. J. Med*. 375 1856–1867. 10.1056/NEJMoa1602252 27718784 PMC5564292

[B13] Garon E. Rizvi N. Hui R. Leighl N. Balmanoukian A. Eder J. (2015). Pembrolizumab for the treatment of non-small-cell lung cancer. *N. Engl. J. Med*. 372 2018–2028. 10.1056/NEJMoa1501824 25891174

[B14] Heneka M. Kummer M. Latz E. (2014). Innate immune activation in neurodegenerative disease. *Nat. Rev. Immunol*. 14 463–477. 10.1038/nri3705 24962261

[B15] Javan M. Aslani S. Zamani M. Rostamnejad J. Asadi M. Farhoodi M. (2016). Downregulation of immunosuppressive molecules, PD-1 and PD-L1 but not PD-L2, in the patients with multiple sclerosis. *Iran J. Allergy Asthma Immunol.* 15 296–302. 27921410

[B16] Ji F. Feng C. Qin J. Wang C. Zhang D. Su L. (2023). Brain-specific Pd1 deficiency leads to cortical neurogenesis defects and depressive-like behaviors in mice. *Cell Death Differ*. 30 2053–2065. 10.1038/s41418-023-01203-3 37553426 PMC10482844

[B17] Jin Y. Hou W. Kang H. Koh C. Kim B. (2013). The expression of PD-1/PDL-1 on CNS cells following Theiler’s virus infection is elevated by IL-6 and inhibited by type I IFN. *J. Immunol.* 190:1. 10.4049/jimmunol.190.Supp.216.7

[B18] Kleinschnitz C. Schwab N. Kraft P. Hagedorn I. Dreykluft A. Schwarz T. (2010). Early detrimental T-cell effects in experimental cerebral ischemia are neither related to adaptive immunity nor thrombus formation. *Blood* 115 3835–3842. 10.1182/blood-2009-10-249078 20215643

[B19] Kummer M. Ising C. Kummer C. Sarlus H. Griep A. Vieira-Saecker A. (2021). Microglial PD-1 stimulation by astrocytic PD-L1 suppresses neuroinflammation and Alzheimer’s disease pathology. *EMBO J*. 40:e108662. 10.15252/embj.2021108662 34825707 PMC8672180

[B20] Linnerbauer M. Beyer T. Nirschl L. Farrenkopf D. Lößlein L. Vandrey O. (2023). PD-L1 positive astrocytes attenuate inflammatory functions of PD-1 positive microglia in models of autoimmune neuroinflammation. *Nat. Commun*. 14:5555. 10.1038/s41467-023-40982-8 37689786 PMC10492803

[B21] Mi Y. Dong J. Liu C. Zhang Q. Zheng C. Wu H. (2024). Amelioration of experimental autoimmune encephalomyelitis by exogenous soluble PD-L1 is associated with restraining dendritic cell maturation and CCR7-mediated migration. *Int. Immunopharmacol*. 143(Pt 2):113398. 10.1016/j.intimp.2024.113398 39423660

[B22] Motzer R. Escudier B. McDermott D. George S. Hammers H. Srinivas S. (2015). Nivolumab versus everolimus in advanced renal-cell carcinoma. *N. Engl. J. Med*. 373 1803–1813. 10.1056/NEJMoa1510665 26406148 PMC5719487

[B23] Qin W. Hu L. Zhang X. Jiang S. Li J. Zhang Z. (2019). The diverse function of PD-1/PD-L pathway beyond cancer. *Front. Immunol*. 10:2298. 10.3389/fimmu.2019.02298 31636634 PMC6787287

[B24] Rosenzweig N. Dvir-Szternfeld R. Tsitsou-Kampeli A. Keren-Shaul H. Ben-Yehuda H. Weill-Raynal P. (2019). PD-1/PD-L1 checkpoint blockade harnesses monocyte-derived macrophages to combat cognitive impairment in a tauopathy mouse model. *Nat. Commun*. 10:465. 10.1038/s41467-019-08352-5 30692527 PMC6349941

[B25] Sharpe A. Pauken K. (2018). The diverse functions of the PD1 inhibitory pathway. *Nat. Rev. Immunol*. 18 153–167. 10.1038/nri.2017.108 28990585

[B26] Shi Y. Ma P. (2024). Pharmacological effects of Astragalus polysaccharides in treating neurodegenerative diseases. *Front. Pharmacol*. 15:1449101. 10.3389/fphar.2024.1449101 39156112 PMC11327089

[B27] Stephenson J. Nutma E. van der Valk P. Amor S. (2018). Inflammation in CNS neurodegenerative diseases. *Immunology* 154 204–219. 10.1111/imm.12922 29513402 PMC5980185

[B28] Takizawa S. Kaneyama T. Tsugane S. Takeichi N. Yanagisawa S. Ichikawa M. (2014). Role of the Programmed Death-1 (PD-1) pathway in regulation of Theiler’s murine encephalomyelitis virus-induced demyelinating disease. *J. Neuroimmunol*. 274 78–85. 10.1016/j.jneuroim.2014.06.018 25027060

[B29] Tan C. Kuchroo J. Sage P. Liang D. Francisco L. Buck J. (2021). PD-1 restraint of regulatory T cell suppressive activity is critical for immune tolerance. *J. Exp. Med*. 218:e20182232. 10.1084/jem.20182232 33045061 PMC7543091

[B30] Tsaktanis T. Linnerbauer M. Lößlein L. Farrenkopf D. Vandrey O. Peter A. (2023). Regulation of the programmed cell death protein 1/programmed cell death ligand 1 axis in relapsing-remitting multiple sclerosis. *Brain Commun*. 5:fcad206. 10.1093/braincomms/fcad206 37564830 PMC10411318

[B31] Wang X. Guo W. Chen Y. Hong C. Ji J. Zhang X. (2024). PD-1/PD-L1 axis is involved in the interaction between microglial polarization and glioma. *Int. Immunopharmacol*. 133:112074. 10.1016/j.intimp.2024.112074 38615383

[B32] Wilson D. Cookson M. Van Den Bosch L. Zetterberg H. Holtzman D. Dewachter I. (2023). Hallmarks of neurodegenerative diseases. *Cell* 186 693–714. 10.1016/j.cell.2022.12.032 36803602

[B33] Wu J. Sun L. Li H. Shen H. Zhai W. Yu Z. (2017). Roles of programmed death protein 1/programmed death-ligand 1 in secondary brain injury after intracerebral hemorrhage in rats: Selective modulation of microglia polarization to anti-inflammatory phenotype. *J. Neuroinflammation* 14:36. 10.1186/s12974-017-0790-0 28196545 PMC5310076

[B34] Yao A. Liu F. Chen K. Tang L. Liu L. Zhang K. (2014). Programmed death 1 deficiency induces the polarization of macrophages/microglia to the M1 phenotype after spinal cord injury in mice. *Neurotherapeutics* 11 636–650. 10.1007/s13311-013-0254-x 24853068 PMC4121443

[B35] Zhao J. Bang S. Furutani K. McGinnis A. Jiang C. Roberts A. (2023). PD-L1/PD-1 checkpoint pathway regulates hippocampal neuronal excitability and learning and memory behavior. *Neuron* 111 2709–2726.e9. 10.1016/j.neuron.2023.05.022 37348508 PMC10529885

[B36] Zhuo Y. Li X. He Z. Lu M. (2023). Pathological mechanisms of neuroimmune response and multitarget disease-modifying therapies of mesenchymal stem cells in Parkinson’s disease. *Stem Cell Res. Ther*. 14:19. 10.1186/s13287-023-03280-0 37041580 PMC10091615

